# Genotypic study of verocytotoxic *Escherichia coli* isolates from deer by multiplex polymerase chain reaction

**DOI:** 10.14202/vetworld.2016.919-921

**Published:** 2016-08-30

**Authors:** Raghavendra Prasad Mishra, Udit Jain, Rakesh Kumar Singh

**Affiliations:** 1 Department of Veterinary Public Health, Veterinary University, UP Pandit Deen Dayal Upadhyaya Veterinary Science University, Mathura, Uttar Pradesh, India; 2 Veterinary Officer, Kanpur Zoo, Kanpur, Uttar Pradesh, India

**Keywords:** deer, polymerase chain reaction, verocytotoxic *Escherichia coli*

## Abstract

**Aim:**

This study was planned to study the genotypes of verocytotoxigenic *Escherichia coli* (VTEC) in fecal samples of deer due to its public health significance.

**Materials and Methods:**

A total of 160 fecal samples of deer were taken from Mathura district and Kanpur Zoo and screened for VTEC genes by polymerase chain reaction (PCR).

**Results:**

All fecal samples were positive for *E. coli*. All the *E. coli* isolates were screened by PCR to detect virulence genes *stx_1_*, *stx_2_*, *eaeA*, and *hlyA*. Of these, 15 isolates were found positive for VTEC having one or more genes in different combinations.

**Conclusion:**

Genes such as *stx_1_*, *stx_2_*, *eaeA*, and *hlyA* were prevalent in VTEC isolates from feces of deer. The presence of VTEC isolates having virulent genes may pose a threat to public health.

## Introduction

Diarrhea is one of the most common and multifactorial diseases of man and animals mainly caused by *Escherichia coli* [1]. *E. coli* is the most commonly observed gastrointestinal flora of animals and environmental contaminant considered as important foodborne pathogen causing serious complications in man and animals [2-5]. Verocytotoxigenic *E. coli* (VTEC) was the first identified as a distinct group of *E. coli* named as verocytotoxigenic *E. coli* (VTEC), which had the ability to produce toxins with profound and irreversible effect on Vero cells. VTEC is also termed as Shiga-like toxin producing *E. coli* or Shiga toxin producing *E. coli* or STEC. Acronym STEC is derived from the fact that the toxins are Shiga like that is similar to those produced by *Shigella dysenteriae* Type 1 [6].

The enterohemorrhagic *E. coli* belongs to the VTEC. VTEC always do not induce clinical signs and are not enterohemorrhagic until addition virulence factor are present like enterohemolysin and adherence factors (intimin). The adherence factor(s) enables the organism to attach to and colonize intestinal mucosal cells [7]. Among VTEC, serotype O157:H7 has been closely associated with the sporadic and clinical outbreaks of hemorrhagic colitis, hemorrhagic uremic syndrome, and thrombotic thrombocytopenic purpura in human beings [8-10].

Healthy domestic ruminants are recognized as the main natural reservoir of STEC and large game animal also be carriers of STEC [11,12]. Keeping in view the importance of this organism, this study was planned to reveal the genotypic study of VTEC in fecal samples of deer.

## Materials and Methods

### Ethical approval

This work does not require ethical approval because we have collected fecal samples of animals after defecation. However, fecal samples were collected without giving any stress or harm to the animals.

### Sampling and isolation of E. coli

A total of 160 samples of deer feces were collected from Mathura district and Kanpur Zoo. The samples were collected aseptically in ultraviolet sterile polythene bags (Fisher Scientific, UK) and immediately transported to the laboratory under chilled conditions for microbiological analysis. For primary isolation of *E. coli* (VTEC), 10 g of fecal sample were enriched in 90 ml modified trypticase soya broth (Hi-media, Mumbai) containing acriflavine 10 mg/L to reduce the growth of Gram-positive organisms. The method used for collection of samples and isolation were made as per the lines suggested by OIE [13]. These samples were incubated at 37°C for 6 h. MacConkey’s Agar was used as differential media, while eosin methylene blue agar (Hi-Media, Mumbai) was used as selective media.

Suspected 160 *E. coli* strains were subjected to morphological, cultural and biochemical characterization as per the standard method [14].

### Molecular characterization

Multiplex polymerase chain reaction (m-pcr) was used for detection of virulent genes (*stx_1_*, *stx_2_*, *eaeA*, and *hlyA*) of 160 *E. coli* isolates. All the 160 *E. coli* isolates were subjected to genomic DNA isolation. The bacterial growth in TSB broth (Hi-Media, Mumbai) was centrifuged at 3000 rpm for 15 min to make the pellet of bacterial cells. These cells were washed twice with PBS (pH 7.4) to remove any impurity of broth media. Bacterial DNA was extracted using DNA extraction kit (Genei, Bangalore) as per the manufacturer’s protocol. PCR master mix solution (Genei, Bangalore) was used. To amplify DNA targeted to virulent genes (*stx_1_*, *stx*_2_, *eaeA*, and *hlyA*) of VTEC using primers on 3 µl of DNA template in 25 µl reaction mixture [15]. After an initial denaturation step at 95°C for 4 min, 30 amplification cycles were performed, each consisting of 94°C for 2 min, 65°C for 2 min, and 72°C for 1.5 min and followed by a final extension step at 72°C for 2.5 min. After the amplification, amplicons were separated in 1.5% gel in tris-acetate ethylenediaminetetraacetic buffer at 60 V for 80 min, stained with 0.5% ethidium bromide solution and visualized under ultraviolet light.

## Results and Discussion

**Figure 1:**
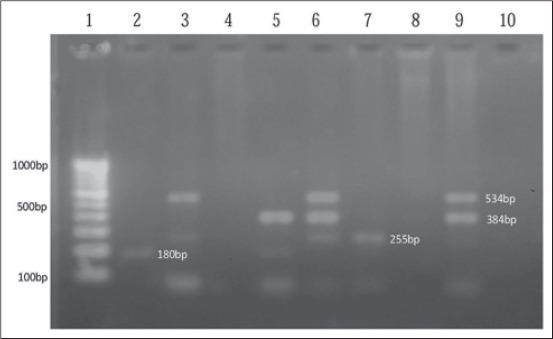
Agarose gel showing PCR amplified product for VTEC gens isolates from faecal sample of Deer. Lane 1:100bp DNA Ladder, Lane 2: *Stx*_1_, Lane 3: *Stx_2_* and *hlyA*, Lane 5: *stx_1_* and *eaeA*, Lane 6: *Stx_2_*, *eaeA* and *hlyA*, Lane 7: *Stx_2_*, Lane 9: *eaeA* and *hlyA*.

All the strains of 160 *E. coli* were screened to detect the presence of VTEC genes using m-PCR (Figure-1). An overall presence of VTEC in deer was found to be 9.37% (15/160). Out of these findings, 4 VTEC 26.6% was found to be positive for *stx_1_* gene (180 bp), 5 VTEC 33.34% was found to be s*tx_2_* gene (255 bp), 3 VTEC 20% was find positive for both genes *stx_1_* and s*tx_2_*, and only one VTEC 6.67% for each positive for *stx_1_* with *eaeA* (384 bp), *stx_2_* with *hlyA* (534 bp) and *stx_2_* with *eaeA* and *hlyA*. In deer previously reported, the prevalence of VTEC was 9.3% and gene *stx_1_* 24%, *stx_2_* 54% and both *stx_1_* with *stx_2_* 5.4% [16], which shows similarity against *stx_1_* gene as present finding. However, the prevalence of *stx_2_* gene in higher level was reported by previous workers as 69.6% [17] and lower finding of *stx_2_* gene 16.2% [18]. The finding of gene *stx_1_* with *stx_2_* was 28.6% [19] which shows a higher prevalence of this gene comparison to present finding and In contrast, 48% of VTEC isolates identified with the *stx_1_* gene only and 40% of STEC isolates with both the *stx_1_* and *stx_2_* genes, respectively [20]. Another lower finding of *stx_1_* gene 11 (16.9%) and higher finding *stx_2_* gene 44 (67.7%) and also lower finding 10 (15.4%) contained both of these genes [21]. Two *E. coli* strains were found having *eaeA* with *hlyA* genes, i.e. lacking *stx* gene as they were enteropathogenic and enterohemmorhagic.

The low infective dose, unusual acid tolerance, and close association with ruminants have made VTEC a serious global zoonotic problem of great public health significance. VTEC can be present in the intestinal tract of a wide range of domestic and wild ruminants (sheep, goats, cattle, buffalo and deer) [22-26].

## Conclusion

The presence of VTEC genes in feces of a large population of deer in different rearing places like zoo and other zoological garden, public health awareness including safe and hygienic practices while handling the deer would be paramount importance in reducing the VTEC infections in humans. Constant monitoring and surveillances program to keep a record of the prevalence from time to time is needed to reduce the chance of infection.

## Authors’ Contributions

UJ designed and planned this research work. RKS helps in collecting the samples to RPM from Kanpur Zoo and RPM executed the isolation and biochemical work. UJ monitored the isolation and biochemical characterization. RPM and UJ were involved in the molecular characterization experiment. The manuscript was drafted and revised by RPM, RKS and UJ. All authors read and approved the final manuscript.
